# Significant association between *TAP2* polymorphisms and rheumatoid arthritis: a meta-analysis

**DOI:** 10.1186/1746-1596-9-129

**Published:** 2014-06-27

**Authors:** Dongjun Dai, Yong Chen, Ping Ru, Xingyu Zhou, Jianmin Tao, Huadan Ye, Qingxiao Hong, Linlin Tang, Guanghui Pan, Danfeng Lin, Qiongyao Gong, Yuelong Lv, Leiting Xu, Shiwei Duan

**Affiliations:** 1 Zhejiang Provincial Key Laboratory of Pathophysiology, School of Medicine, Ningbo University, Ningbo, Zhejiang 315211, China; 2 Department of Rheumatology, Ningbo No.2 Hospital, Ningbo, Zhejiang 315010, China

**Keywords:** Rheumatoid arthritis, Polymorphism, Meta-analysis, TAP2, Ethnicity

## Abstract

**Background:**

Rheumatoid arthritis (RA) is a severe chronic immune mediated inflammatory disease that has been shown to be associated with human leukocyte antigen (HLA) loci. The transporter associated with antigen processing 2 (*TAP2*) has been identified to play an important role in the HLA-associated diseases and immune response. The goal of our meta-analysis was to summarize the contribution of *TAP2* polymorphisms to the risk of RA.

**Methods:**

Meta-analyses were performed between RA and 3 *TAP2* coding polymorphisms that comprised *TAP2*-379Ile > Val (rs1800454), *TAP2*-565Ala > Thr (rs2228396) and *TAP2*-665Thr > Ala (rs241447). The meta-analyses were involved with 9 studies (24 individual studies) among 973 cases and 965 controls.

**Results:**

Meta-analyses showed that *TAP2*-379Ile allele was significantly associated with an increased risk of RA (*p* = 0.0002, odds ratio (OR) = 1.44, 95% confidence interval (CI) = 1.18-1.74). This association was further shown only in the dominant model (*p* = 0.006, OR = 1.59, 95% CI = 1.14-2.22). Subgroup analyses by ethnicity revealed that the association of *TAP2*-379Ile was significant in Asians (*p* = 0.03, OR = 1.38, 95% CI = 1.04-1.83). In addition, another significant association of *TAP2*-565Thr allele with RA was observed in Europeans (*p* = 0.002, OR = 1.62, 95% CI = 1.20-2.20).

**Conclusions:**

Our meta-analyses suggested that *TAP2*-379Ile allele was significantly associated with a 59% increased risk in the dominant effect model. Subgroup analyses by ethnicity showed that *TAP2*-379-Ile increased the risk of RA by 38% in Asians and *TAP2*-565Thr increased the risk of RA by 38% in Europeans.

**Virtual Slides:**

The virtual slide(s) for this article can be found here: http://www.diagnosticpathology.diagnomx.eu/vs/2097080313124700

## Background

Rheumatoid arthritis (RA) is a common immune-mediated chronic inflammatory disease [1]. Severe clinical symptoms of RA comprise bone lose [2] and heart diseases [3] that can make a huge destruction to human body. RA is a complex disease caused by both genetic and environmental factors. Twin studies estimated a large heritability (60%) in RA [4]. Family-based studies also demonstrated that genetic factors played a more important role in the development of RA than environmental factors [5,6].

Consistent association was found between human leukocyte antigen (HLA) loci and RA [7]. The transporter associated with antigen processing 2 (*TAP2*) gene encodes transporter 2, ATP-binding cassette, sub-family B (MDR/TAP) that is a major histocompatibility complex (MHC) gene located between HLA-DP and HLA-DQ [8]. TAP2 delivers antigenic peptides to the endoplasmic reticulum of HLA class I molecular [9], especially in selecting the size of peptides [10]. *TAP2* has been shown to play an important role in the HLA-associated diseases and immune response [11].

*TAP2* polymorphisms have been tested for their association with the occurrence and development of RA [12-23]. Among them, Ile379Val, Ala565Thr and Thr665Ala are most often studied [14-23]. Altogether there were 10 case–control studies for the *TAP2* polymorphisms with RA, yielding only 2 significant results (Table 1). We suspected that a lack of power in the previous studies with moderate sample size might influence the reliability of the results [24]. Meta-analysis is often used to enhance statistical power and thus is likely to produce a more convincing conclusion [25]. Here we performed a set of meta-analyses of the three polymorphisms by pooling up the data from individual association study [25]. Our research is likely to provide a better evaluation of the contribution of *TAP2* polymorphisms to the risk of RA.

**Table 1:** Characteristics of the case–control studies in the current meta-analyses

**Gene locus**	**First author**	**Year**	**Country**	**Ethnicity**	**Genotyping method**	**Cases/controls**	**Control source**	**HWE**	Result*	**Power**	**MAF**
*TAP2*-379	B. P. Wordsworth [14]	1993	Britain	Europeans	ARMS-PCR	60/117	Population	NA	NS	0.101	0.167
	Sara Marsal [15]	1994	America	Europeans	ARMS-PCR	185/48	Population	NA	NS	0.086	0.177
	M.C. Hillarby [16]	1996	Britain	Europeans	ARMS-PCR	89/64	Population	NA	S	0.091	0.172
	F. Takeuchi [17]	1997	Japan	Asians	PCR-RFLP	92/95	Population	NA	NS	0.095	0.132
	J Vinasco [18]	1998	Spain	Europeans	PCR-RFLP	50/55	Population	NA	NS	0.072	0.109
	Sasijit Vejbaesya [19]	2000	Thailand	Asians	ARMS-PCR	82/100	Population	Yes	NS	0.115	0.220
	S.-L. Zhang [20]	2002	France	Europeans	ARMS-PCR	138/100	Population	Yes	NS	0.098	0.120
	Min-Chien Yu [21]	2004	China	Asians	PCR-RFLP	100/99	Population	Yes	NS	0.124	0.237
*TAP2*-565	B. P. Wordsworth [14]	1993	Britain	Europeans	ARMS-PCR	60/117	Population	NA	NS	0.094	0.132
	Sara Marsal [15]	1994	America	Europeans	ARMS-PCR	185/48	Population	NA	NS	0.081	0.156
	M.C. Hillarby [16]	1996	Britain	Europeans	ARMS-PCR	89/64	Population	NA	NS	0.086	0.148
	F. Takeuchi [17]	1997	Japan	Asians	PCR-RFLP	92/95	Population	NA	S	0.09	0.116
	J Vinasco [18]	1998	Spain	Europeans	PCR-RFLP	50/55	Population	NA	NS	0.059	0.036
	S.-L. Zhang [20]	2002	France	Europeans	ARMS-PCR	138/100	Population	Yes	S	0.078	0.070
	Min-Chien Yu [21]	2004	China	Asians	PCR-RFLP	100/99	Population	Yes	NS	0.086	0.096
	Yu L [22]	2013	China	Asians	PCR-RFLP	177/288	Population	Yes	NS	0.169	0.142
*TAP2*-665	B. P. Wordsworth [14]	1993	Britain	Europeans	ARMS-PCR	60/117	Population	NA	NS	0.115	0.248
	Sara Marsal [15]	1994	America	Europeans	ARMS-PCR	185/48	Population	NA	NS	0.084	0.167
	M.C. Hillarby [16]	1996	Britain	Europeans	ARMS-PCR	89/64	Population	NA	NS	0.119	0.375
	F. Takeuchi [17]	1997	Japan	Asians	PCR-RFLP	92/95	Population	NA	NS	0.136	0.347
	J Vinasco [18]	1998	Spain	Europeans	PCR-RFLP	50/55	Population	NA	NS	0.091	0.264
	Juha Tuokko [23]	1998	Finland	Europeans	PCR-RFLP	40/60	Population	No	NS	0.095	0.350
	S.-L. Zhang [20]	2002	France	European	ARMS-PCR	138/100	Population	Yes	NS	0.148	0.300
	Min-Chien Yu [21]	2004	China	Asians	PCR-RFLP	100/99	Population	Yes	NS	0.147	0.601*

ARMS-PCR: Amplification refractory mutation system; PCR-RFLP: Polymerase Chain Reaction –Restriction Fragment Length Polymorphism; HWE.

Hardy-Weinberg equilibrium; Result*, The association between IL1B gene and PD; NS, No significant; S, Significant; NA: Not applicable; *The reference allele is TAP2-665Ala.

## Methods

We collected studies that examined the associations of *TAP2* polymorphisms with RA in September of 2013 by searching the online databases (PubMed, WanFang, WeiPu and CNKI) without time and language restriction, using the keywords “rheumatoid arthritis *TAP2* association” and “rheumatoid arthritis *TAP2* polymorphism”. The obtained studies would be included in our meta-analyses if they met the following criteria: (1) It was an original case–control study with an assessment of the association of *TAP2* with RA in humans; (2) It contains sufficient information to infer the odds ratios (ORs) and 95% confidence intervals (95% CIs); (3) Genotype distribution of each polymorphism in controls met Hardy-Weinberg equilibrium (HWE); (4) The cumulative number of individual studies for one genetic locus are at least three. We extracted or calculated the following information from each selected study: the first author, year of publication, country, ethnicity, genotyping method, numbers of cases and controls, control source, HWE for controls, reported association results, power of each involved study and minor allele frequency (MAF) in each stage.

Since some studies presented the data of haplotypes (A to H, Table 2), our study translated the haplotypes into the genotypes of three coding polymorphisms (*TAP2*-379, *TAP2*-565, and *TAP2*-665) [8,26]. We used Arlequin program [27] to test whether the genotyping distribution in controls was in HWE. Cochran’s Q statistic and I^2^ test [28] were used to calculate statistical heterogeneity. Fixed-effect model would be used to the studies with minimal to moderate heterogeneity (I^2^ < 50%), and the random-effect model would be used to the studies with significant heterogeneity (I^2^ > =50%). Combined ORs and CIs were estimated by Review Manager 5 [29]. Funnel plots were drawn to observe the potential publication bias. The power of each study was calculated by Power and Sample Size Calculation program. All the statistical analyses were performed by two independent reviewers (Ping Ru and Xingyu Zhou).

**Table 2:** Nomenclature of TAP2 haplotypes

**TAP2**	**TAP2-379**		**TAP2-565**		**TAP2-665**	
	**GTA**	**ATA**	**GTA**	**ATA**	**GTA**	**ATA**
A	Val		Ala		Thr	
B	Val		Ala			Ala
C		Ile	Ala		Thr	
D		Ile		Thr	Thr	
E	Val			Thr	Thr	
F	Val		Ala		Thr	
G		Ile	Ala			Ala
H		Ile		Thr		Ala

Val: Valine; Ile: Isoleucine; Ala: Alanine; Thr: Threonine.

## Results and discussion

**Figure 1:**
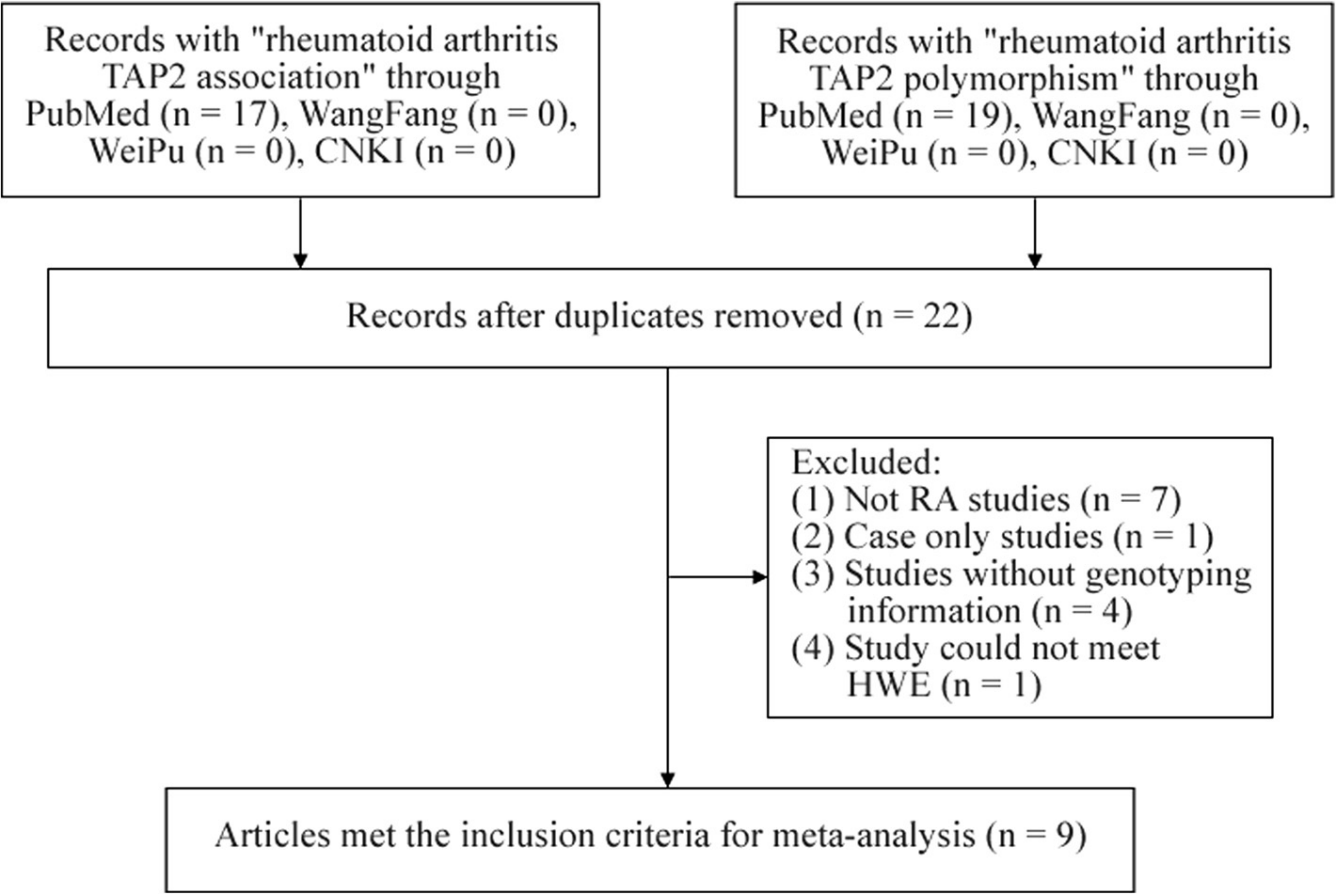
Flowchart of selection process in the meta-analyses.

As shown in Figure 1, 22 genetic studies on *TAP2* gene were initially collected. Of them, we excluded 7 studies that were not related to RA, 1 case-only study, 4 studies without genotyping information, 1 study that did not meet HWE. Finally, 9 articles [14-22] were involved in the current study. Altogether, there were 973 RA patients and 965 controls in the meta-analyses of 3 *TAP2* polymorphisms (Tables 1 and 3).

**Table 3:** Meta-analyses of TAP2-379, TAP2-565, TAP2-665with RA*

**Genetic locus**	Cases/controls (S*)	**Genetic model**	**Ethnicity**	**OR (95% CI)**	***P *** **value**	**I**^**2**^	**Power**
*TAP2*-379	796/677 (8)	Overall ( I vs V)	Overall	1.44 (1.18-1.74)	0.0002	42%	0.477
	522/768 (5)		Europeans	1.39 (0.88-2.20)	0.16	64%	0.286
	274/293 (3)		Asians	1.38 (1.04-1.83)	0.03	0%	0.241
	320/298 (3)	Dominant ( II/IV vs VV )		1.59 (1.14-2.22)	0.006	0%	0.336
	320/298 (3)	Recessive ( II vs IV/VV )		1.24 (0.58-2.64)	0.58	0%	0.104
	198/209 (3)	Additive ( II vs VV )		1.49 (0.68-3.23)	0.32	0%	0.101
*TAP2*-565	891/871 (8)	Overall ( T vs A )	Overall	1.23 (0.84-1.81)	0.28	64%	0.439
	522/778 (5)		Europeans	1.62 (1.20-2.20)	0.002	24%	0.229
	369/482 (3)		Asians	0.87 (0.43-1.75)	0.70	76%	0.254
	415/487 (3)	Dominant ( TT/TA vs AA )		1.17 (0.85-1.61)	0.33	45%	0.379
	415/487 (3)	Recessive ( TT vs TA/AA )		4.71 (0.51-43.33)	0.17	64%	0.075
	335/384 (3)	Additive ( TT vs AA )		4.64 (0.52-41.77)	0.17	64%	0.075
*TAP2*-665	714/578 (7)	Overall ( A vs T )	Overall	1.02 (0.85-1.22)	0.84	48%	0.557
	522/384 (5)		Europeans	0.87 (0.69-1.09)	0.23	39%	0.475
	192/194 (2)		Asians	1.32 (0.99-1.77)	0.06	0%	0.080
	238/199 (2)	Dominant ( AA/AT vs TT )		1.07 (0.44-2.57)	0.88	67%	0.252
	238/199 (2)	Recessive ( AA vs AT/TT )		0.58 (0.06-5.77)	0.64	77%	0.131
	128/100 (2)	Additive ( AA vs TT )		1.15 (0.53-2.49)	0.73	83%	0.122

S*: Amount of stages; V: Valine; I: Isoleucine; A: Alanine; T: Threonine.

**Figure 2:**
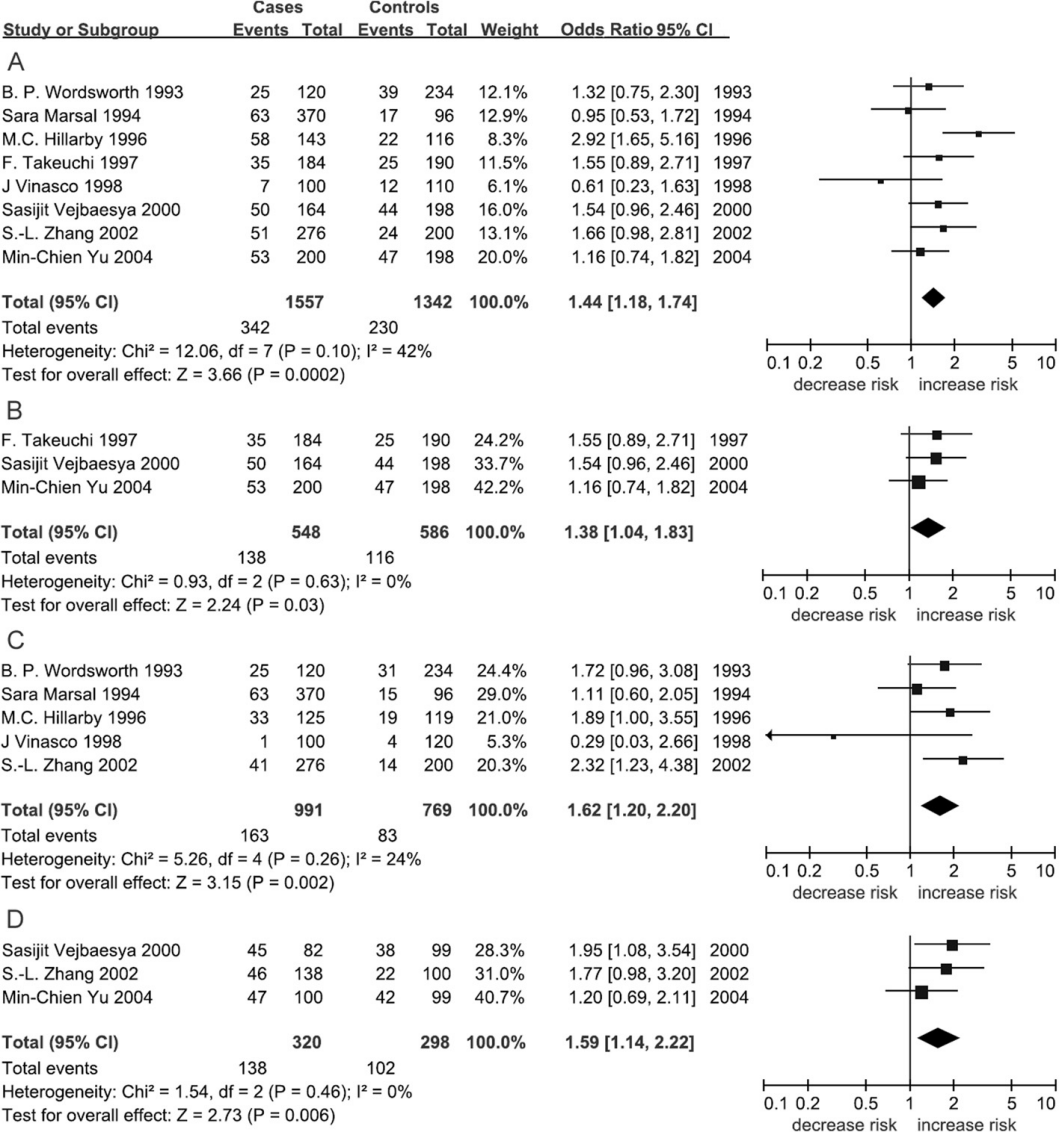
**Forest plots of TAP2 gene polymorphisms with RA. A**: Forest plot of *TAP*-379 with RA for combined population; **B**: Forest plot of *TAP*-379 with RA in Asians; **C**: Forest plot of *TAP*-379 with RA under dominant effect model; **D**: Forest plot of *TAP*-565 with RA in Europeans.

In the present study, we tested the associations between 3 *TAP2* polymorphisms and RA disease. Different inheritable models, including dominant, recessive and additive models, were also tested for all 3 polymorphisms. As shown in Table 3 and Figure 2, a significant association of *TAP2*-379Ile with increased risk of RA was found for combined population (*p* = 0.0002, OR = 1.40, 95% CI = 1.16-1.70). Subgroup analysis by ethnicity showed that this significant association was only found in Asians (*p* = 0.03, OR = 1.38, 95% CI = 1.04-1.83, Table 3 and Figure 2) but not in Europeans (*p* = 0.16, Table 3). In addition, significant contribution of *TAP2*-379Ile to RA was only found in the dominant model (*p* = 0.006, OR = 1.59, 95% CI = 1.14-2.22, Table 3, Figure 2). For *TAP2*-565Thr, there was no significant result in the combined populations (*p* = 0.28, Table 3), but we found significant association between Europeans and RA (*p* = 0.002, OR = 1.62, 95% CI = 1.20-2.20, Table 3, Figure 2). For *TAP2*-665 locus, no significant result was found in the combined meta-analyses or in the subgroup meta-analyses (Table 3).

Significant statistical heterogeneity was found in the meta-analyses of *TAP2*-565 (I^2^ = 64%). A further subgroup meta-analyses by ethnicity showed that significant heterogeneity was coming from the Europeans for *TAP2*-379 (I^2^ = 64%) and Asians for *TAP2*-565 (I^2^ = 76%). Further subgroup studies showed that significant heterogeneity also existed in the meta-analyses of *TAP2*-565 (recessive model: I^2^ = 64%; additive model: I^2^ = 64%) and *TAP2*-665 (dominant model: I^2^ = 67%, recessive model: I^2^ = 77%; additive model: I^2^ = 83%).

**Figure 3:**
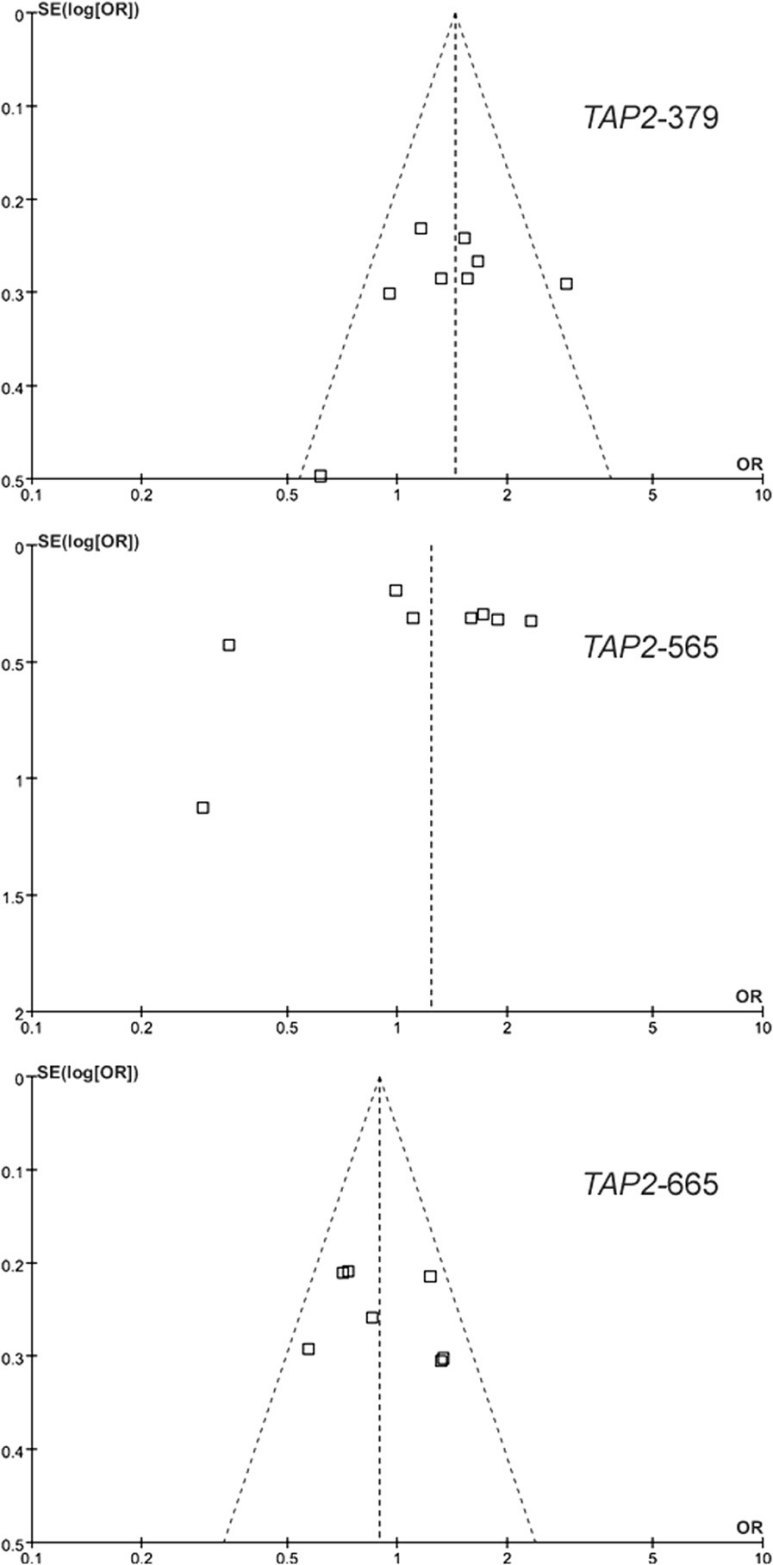
Funnel plots of TAP2 gene polymorphisms with RA.

All the power analyses in current meta-analyses were tested under a moderate risk of SCZ (OR = 1.2, Tables 1 and 2). Compared with previous individual studies, our meta-analyses showed a much stronger power (Tables 1 and 3). In addition, no publication bias for the meta-analyses was observed (Figure 3).

To our knowledge, our study was the first meta-analyses of the three coding polymorphisms of *TAP2* gene. Previous studies showed significant association between *TAP2* gene and autoimmune diseases such as allergic rhinitis [30], systemic lupus erythematosus [31] and RA [20,21]. Our results indicated that *TAP2*-379Ile was able to increase 44% of the risk of RA in all subjects and 38% of the risk of RA in Asians. Moreover, *TAP2*-379Ile was associated with a 59% increased risk of RA in the dominant model. We also found that *TAP2*-565Thr increased the risk of RA by 62% in Europeans.

Previous RA association studies observed a handful of single nucleotide polymorphisms (SNPs) with dominant effect such as -607A/C polymorphism of *IL-18* gene [32], rs10489629 of *IL-23R* gene [33], -173G/C polymorphism of *MIF* gene [34] and -607A/C polymorphism of *IL-18* gene [32]. Our observation of significant association of *TAP2*-379Ile polymorphism in the dominant model supported that dominant model might be a key genetic model in the pathogenesis of RA disease.

Since MAFs are different in different populations, we further evaluated the role of *TAP2* polymorphisms in Asians and Europeans separately. For *TAP2*-379Ile, significant association was found in Asians but not in Europeans. On the contrary, we observed *TAP2*-565Thr as a risky factor of RA in Europeans but not in Asians. This might be due to a lack of power in the subgroup meta-analysis in Europeans for *TAP2*-379 (power = 0.286, I^2^ = 64%) and in Asians for *TAP2*-565 (power = 0.254, I^2^ = 76%). To be noted, one Asian study [21] involved in the meta-analyses of *TAP2*-665Ala was found a high allele frequency (allele frequency = 0.601, Table 1) than those in the rest studies (average allele frequency = 0.284).

Our meta-analyses presented several limitations that needed to be taken with cautions. Firstly, the associations of *TAP2* polymorphisms were only evaluated in Europeans and Asians. The findings might not be feasible for other populations such as Africans. And potential difference in intra-European and intra-Asian population might influence the result of our study (see the MAFs in Table 1). Secondly, RA is a complex chronic disease. Different clinical variables may influence the results of the current study. Hidden physiological factors may exist in the RA patients and affect the quality of the current meta-analysis. Further studies with precise diagnosis might be helpful for a better meta-analysis in the future. Thirdly, although the power of current meta-analyses was much stronger than the previous studies, more replicated studies are required to strengthen the stability of the association between *TAP2* polymorphisms and RA. Fourthly, certain multiple testing existed in the current study, and cautions needed to be taken for the significant results. Fifthly, there are 2232 polymorphisms in *TAP2* gene according to the NCBI dbSNP database. Our study only focused on three polymorphisms of *TAP2* that might be hard to give fully consideration of the contribution of *TAP2* polymorphisms. Moreover, the 3 *TAP2* polymorphisms might not be the causal variants but be in high linkage disequilibrium with other established RA MHC variants. Sixthly, since significant associations were found in the both allelic and dominant model for *TAP2*-379, our results implied the genetic models of *TAP2*-379 polymorphism were complex and hard to be determined. Seventhly, there were publication biases for the current meta-analyses of *TAP2*-379 and *TAP2*-565. After removing the outlier studies [16,17], the results of the two meta-analyes remained the same as the previous one.

## Conclusions

In summary, our meta-analyses suggested that *TAP2*-379Ile allele was significantly associated with a 59% increased risk in the dominant model. To be more specifically, *TAP2*-379-Ile increased the risk of RA by 38% in Asians and *TAP2*-565Thr increased the risk of RA by 38% in Europeans. Future large-scale and well designed studies are required to confirm our findings and to reveal other *TAP2* polymorphisms with contribution to RA disease.

## Abbreviations

RA: Rheumatoid arthritis; SNPs: Single nucleotide polymorphisms; HWE: Hardy-Weinberg equilibriu; ORs: Odds ratios; 95% CIs: 95% confidence intervals.

## Competing interests

None of the authors have any commercial or other association that might pose a conflict of interest. All authors are responsible for the content and writing of the paper.

## Authors’ contributions

SD participated in research design. YC, PR, XZ, YH and LX selected the articles, QH, LT, GP, DL, QG and YL performed data analysis. The manuscript was drafted by DD, YC and SD, and critically reviewed and discussed with the other co-authors. All the authors read and approved the final manuscript.

## Authors’ information

Dongjun Dai and Yong Chen: Co-first authors of this work.
